# Growth Rate Consequences of Coloniality in a Harmful Phytoplankter

**DOI:** 10.1371/journal.pone.0008679

**Published:** 2010-01-13

**Authors:** Alan E. Wilson, RajReni B. Kaul, Orlando Sarnelle

**Affiliations:** 1 Department of Fisheries and Allied Aquacultures, Auburn University, Auburn, Alabama, United States of America; 2 Department of Fisheries and Wildlife, Michigan State University, East Lansing, Michigan, United States of America; Mt. Alison University, Canada

## Abstract

**Background:**

Allometric studies have shown that individual growth rate is inversely related to body size across a broad spectrum of organisms that vary greatly in size. Fewer studies have documented such patterns within species. No data exist directly documenting the influence of colony size on growth rate for microscopic, colonial organisms.

**Methodology/Principal Findings:**

To determine if similar negative relationships between growth rate and size hold for colonial organisms, we developed a technique for measuring the growth of individual colonies of a bloom-forming, toxic cyanobacterium, *Microcystis aeruginosa* using microscopy and digital image analysis. For five out of six genotypes of *M. aeruginosa* isolated from lakes in Michigan and Alabama, we found significant negative relationships between colony size and growth rate. We found large intraspecific variation in both the slope of these relationships and in the growth rate of colonies at a standard size. In addition, growth rate estimates for individual colonies were generally consistent with population growth rates measured using standard batch culture.

**Conclusions/Significance:**

Given that colony size varies widely within populations, our results imply that natural populations of colonial phytoplankton exist as a mosaic of individuals with widely varying ecological attributes (since size strongly affects growth rate, grazing mortality, and migration speed). Quantifying the influence of colony size on growth rate will permit development of more accurate, predictive models of ecological interactions (e.g., competition, herbivory) and their role in the proliferation of harmful algal blooms, in addition to increasing our understanding about why these interactions vary in strength within and across environments.

## Introduction

Understanding how physiological and ecological processes scale with organism size has important implications for elucidating the mechanisms structuring organisms, populations, communities, and whole ecosystems [1], [2]. Consequently, there has been much interest in describing broad-scale allometric relationships spanning individual cells to megafauna across up to 27 orders of magnitude in mass [3]. Using the power function: *R = aM^b^* (where *R* is the physiological rate of interest, *M* is organism mass, and *a*, *b* are scaling constants), scaling of these relationships has been shown to vary across species, but *b* tends to be near a multiple of 1/3 or 1/4 [4], [5]. Given the interspecific nature of most allometric datasets, existing relationships between size and physiological/ecological attributes are confounded, at least to some degree, by the little known influence of species. The relatively few studies that have documented intraspecific allometric variation have shown departures from the universal patterns (reviewed by [6]). Some argue that such intraspecific variation in *b* is simply a consequence of a narrowed range in organism sizes [3], [7]. Such intraspecific variation could be ecologically important [6], but we have no way of assessing this given the limited number of studies that quantify allometric relationships within species.

Given the extremely wide size range of phytoplankton (∼seven orders of magnitude by mass, [8]) as well as their global importance as primary producers, there has been significant theoretical [9], [10] and empirical [11], [12], [13], [14] interest in understanding how phytoplankton size influences algal physiology and ecology (reviewed by [15], [16]). Phytoplankter cell size has been shown to be positively related to chlorophyll *a* content [17], [18], macronutrient content [13], [19], and sinking speed [20], [21], while typically being negatively related to growth rate ([12], [17]; but see [13], [22]). Studies examining similar ecophysiological patterns for colonial phytoplankton species are exceedingly rare [23], [24]. There is also evidence that large size can afford protection against zooplankton grazing ([25], [26]; but see [27], [28]), photoinhibition [29], or viral infection [30]. With the exception of a few studies on diatoms [12], [14], [31] or macroscopic benthic cyanobacteria (*Nostoc* spp.; [32], [33], [34]), past empirical analyses of the influence of algal size have focused on patterns across species [8], [23], [35], [36]. To our knowledge, no study has examined how colony size affects growth rate of individual colonies within a phytoplankton species. This lack of knowledge may in part reflect methodological challenges associated with quantifying growth rates of individual colonies of microscopic organisms.

Cyanobacteria are a major threat to global drinking water supplies [37], [38], [39]. Of the bloom-forming cyanobacteria, *Microcystis aeruginosa*, is one of the most widely-studied in part because of the potent liver toxin (microcystin) it may produce [37], [38], [39], [40]. In nature, *M. aeruginosa* grows as mucilaginous colonies typically harboring many thousands of cells [41], [42], and recent studies indicate that a single genotype may exhibit various colony morphologies under different environmental conditions [43]. Colony formation in *Microcystis* is not well understood, but available data suggest that small, overwintering colonies enter the water column from the sediment early in the Spring [44]. Through cell division, the colonies grow until they get large and fragile whereby they break into smaller colonies. This cycle is repeated through the growing season until the colonies return to the sediments in the cooler months. In addition to large variation in colony morphology, recent experiments have shown large variability in growth rate, toxin quota, and colony size among genotypes of *M. aeruginosa* [45], [46], [47]. Large colony size should reduce *M. aeruginosa* mortality from zooplankton grazing [28], [48], in addition to allowing for greater vertical velocities in this buoyancy-regulating species [8]. These advantages may come at a cost, however, in terms of reduced growth rate, but this cost has yet to be measured for any colonial microbe. We would expect a negative relationship between growth rate and colony size for *M. aeruginosa* given functional constraints (e.g., nutrient diffusion limitations) associated with the reduced surface-to-volume ratio of larger colonies and the fact that the cells of *M. aeruginosa* are distributed throughout a three-dimensional colony matrix (rather than being restricted to the outer surface of the colony as in *Volvox*, for example). Quantifying the influence of colony size on growth rate is crucial for the development of predictive models aimed at forecasting blooms of colonial cyanobacteria, as well as for a more general understanding of phytoplankton dynamics.

In this paper, we describe a simple technique for estimating the growth rate of individual phytoplankton colonies that enabled us to quantify relationships between growth rate and colony size within six genotypes of *M. aeruginosa* collected from lakes in Michigan and Alabama. We tested the hypothesis that growth rate declines with initial colony size and examined whether the influence of colony size on growth rate varied across genotypes. Additionally, two critical methodological issues were examined. First, a potential artifact stemming from greater nutrient uptake by larger colonies in small experimental chambers was tested by comparing the growth rates of small *M. aeruginosa* colonies grown in isolation versus with a large colony. Second, growth rates of individual *M. aeruginosa* colonies measured using our individual colony approach were compared to population growth estimates in standard batch culture.

## Methods

The method for measuring the growth of individual phytoplankton colonies we developed consists of isolating single colonies into wells of a chambered microscope slide and measuring the volume of the colony over time using an image analysis system and a dissecting microscope. Growth rate is then calculated from changes in colony volume over several days. Chambered slides consist of a clear, polystyrene 8-well chamber attached to a standard glass microscope slide with a clear, polystyrene cover (NUNC Lab-Tek II Chamber Slide System, part# 154534). Individual wells are 11 mm (height)×11 mm (width)×9 mm (depth), with a total volume of ∼1 ml. All chambers used in this study were new and sterile. Before inoculating single colonies into individual wells of each chamber, 500 µl of sterile algal medium (modified WC medium, 500 µM NH_4_Cl, 25 µM K_2_HPO_4_, no Si; [49]) was added to each well and the chambers were exposed to strong UV light for 15 minutes to reduce the probability of contamination. All colony transfers were performed in a UV-sterilized, laminar flow hood.

In the first experiment, we quantified growth rate-size relationships for three *M. aeruginosa* genotypes isolated from three hard-water Michigan lakes that varied widely in trophic status (Hudson Lake, Magician Lake, Swan Lake; summer total phosphorus range = 25–87 µg L^−1^, all isolated in 2002; [50]). The *M. aeruginosa* genotypes have previously been shown to vary in population growth rate in batch culture (range = 0.20–0.39 day^−1^; [47]), but it is not clear whether these differences were driven by differences in colony size or other factors that vary across genotypes. All genotypes were maintained in the laboratory in modified WC medium. Experimental conditions were: 24°C, 60 µmol photons m^−2^ s^−1^, 16 h∶8 h light∶dark cycle, and pH 7.22. Before the experiment, batch cultures of each genotype were acclimated to experimental conditions of nutrients and light for four weeks in 150 ml flasks filled with 100 ml of medium. At the start of the experiment, a 1 ml subsample from a batch culture was pipetted into a sterile petri dish filled with 5 ml of fresh, sterile medium. A single colony was then inoculated into each well (containing 500 µl of sterile medium) of an 8-welled slide, such that small and large colonies of all three genotypes were equally represented on every slide. Slides were rotated to a different location in the incubator once daily to homogenize light intensities across slides.

**Figure 1:**
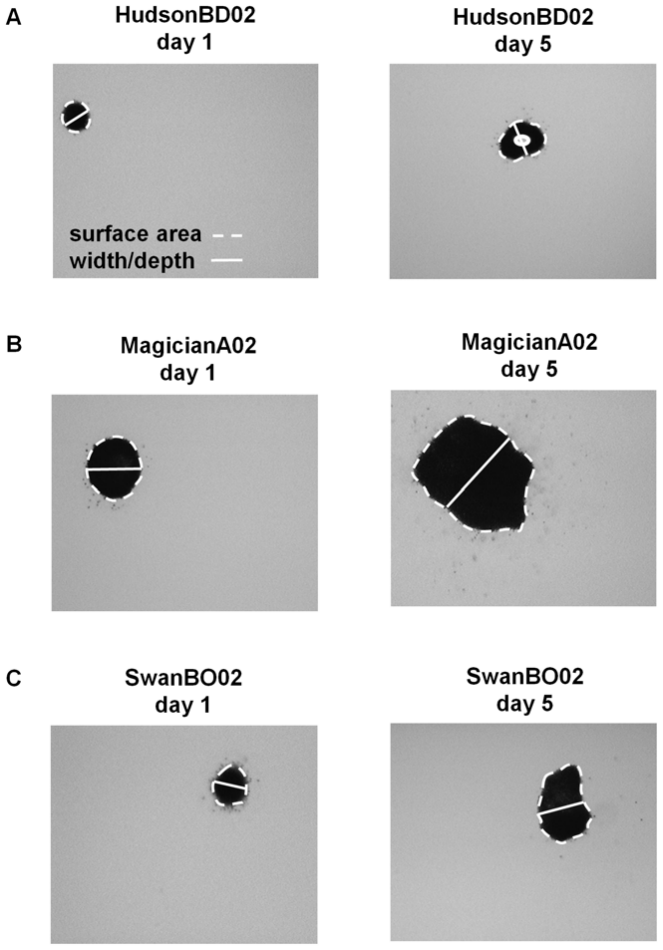
*Microcystis aeruginosa* colony size measurements. The surface area and width of individual colonies was measured using microscopy and digital image analysis. Example measurements for individual colonies of three *M. aeruginosa* genotypes ((A) HudsonBD02, (B) MagicianA02, and (C) SwanBO02) measured on days 1 and 5. Stippled white line was traced with a mouse for estimation of surface area. Solid white line represents our approximation of colony depth (see text for explanation). For HudsonBD02 (A), the perimeter of the void in the center of the colony was also traced and its estimated surface area was subtracted from the total surface area. All photos taken at 63× magnification.

Colonies of *M. aeruginosa* exhibit a tremendous variety of three-dimensional shapes (although the smallest colonies tend to be roughly spherical), so volume estimates cannot be obtained from simple measurements of linear dimensions. We measured colonies under a dissecting microscope (magnifications ranged from 20x to 63x) using images captured with a digital camera. Before an image was captured, each colony was first positioned in the center of the well using a sterile pipette tip so that the colony's largest surface area dimension was horizontal. From each image, the surface area (mm^2^) of each colony was measured using ImagePro Plus (2004) software calibrated for each magnification. Colonies sometimes have spaces within them that are devoid of cells (4% of the colonies used in this study had voids, Fig. 1A). The surface areas of these voids were estimated as above and subtracted from the estimate of colony surface area.

To calculate colony volume, we needed an estimate of depth for each colony, which we assumed was approximately equal to the width of the colony measured perpendicular to the greatest axial linear dimension near the middle of the colony (Fig. 1). Observations of rotated colonies indicated that this approach provided a reasonable approximation of colony depth. We calculated colony volume (mm^3^) as the product of surface area and depth. Given that small colonies were more likely to be spherical than large colonies, our approach for calculating colony volume may overestimate volumes for small colonies. This minor bias had no measureable influence on the relationships between colony size and growth rate. We validated our measurement approach indirectly by comparing growth rates of measured colonies to growth rates of batch cultures (see below).

We measured the volumes of colonies on days 1, 4, and 8 of the first experiment. We chose an 8-day incubation based on previous observations that batch cultures of the *M. aeruginosa* genotypes used in this paper grow exponentially over 8 days under similar nutrient and light conditions [47], and the need for substantial differences in colony volume to occur between measurements in order to obtain precise estimates of growth rate. Colony growth rates (*r*, assuming exponential growth) were calculated by fitting linear regressions to the natural log of colony volume versus time (*t*) and calculating the regression slope: ln*V_t_* = ln*V_0_* + *rt*.

In the second experiment, we quantified growth rate-size relationships for four *M. aeruginosa* genotypes that were collected from lakes in southern Michigan (Hudson Lake and Swan Lake, 2002 isolation), Alabama (2008 isolation), and Lake Erie (2006 isolation), while examining a potential bias resulting from differential resource exhaustion in the growth chambers by colonies of different size. Large colonies, by virtue of their higher rates of resource uptake, might experience greater resource limitation over time in 500 µl of medium than small colonies, which could bias the results to show slower growth by larger colonies. To rule out this bias, we compared the growth rate of small colonies under two conditions: in isolation and when paired with a large colony. If large colonies significantly deplete resources in the growth chambers, the growth of small colonies should be lower when paired with a large colony.

For the second experiment, all genotypes were maintained in the laboratory in 150 ml flasks under experimental conditions (24°C, 45 µmol photons m^−2^ s^−1^, 16 h∶8 h light∶dark cycle, and WC medium at pH 7.14) for 47 days prior to the experiment. The experiment consisted of eight replicates of two treatments – a small colony grown in isolation (control) or a small colony grown with a large colony of the same genotype (treatment) – distributed across eight chambered slides (1 treatment well genotype^−1^ chamber^−1^). After previewing colonies from each batch culture, small or large colonies were selected based on their distribution across the size gradient. Initial colony volume for the small colonies did not vary between treatments for any of the *M. aeruginosa* genotypes (Analysis of variance (ANOVA), *P* >0.53). Colony volume (*V_t_*) was measured on days 1 and 7 using the same methods as described for the first experiment, and growth rate was calculated as: *r* = ln(*V_7_*/*V_1_*)/*t*. Analysis of variance (ANOVA) was used to determine if mean growth rate of each genotype varied between small colonies grown in isolation or with a large colony. One Hudson Lake control replicate did not grow and was not included in analyses (*n* = 7).

As a means of validating the entire protocol for measuring individual colony growth, we compared the growth of individual *M. aeruginosa* colonies in chambered slides to growth measured by a conventional batch culture approach in experiment 3. This experiment was conducted using the three genotypes from the first experiment, plus genotypes from two other southern Michigan lakes (Gilkey Lake, Gull Lake; summer total phosphorus range = 17–20 µg L^−1^; [50]). Experimental conditions and methods for measuring colony volume were the same as in the first experiment, except that each genotype was acclimated under experimental conditions for 38 days. Eight replicate colonies per genotype were distributed across six chambered slides (1 or 2 treatment wells genotype^−1^ chamber^−1^). Colonies used in this experiment were selected to be representative of the range of colony size in each culture, and colony volume (*V_t_*) was measured on days 1 and 5. Colony growth rates were calculated as: *r* = ln(*V_5_*/*V_1_*)/*t*. One individual colony replicate from Magician Lake was lost during the experiment (*n* = 7).

Population growth was measured simultaneously in batch cultures: 8 replicate, 150 ml flasks filled with 100 ml of sterile WC medium were inoculated with 1 ml of each of the five genotypes to start the experiment. Flasks and chambered slides were incubated side-by-side in the same incubator and were rotated within the incubator daily to homogenize light levels. Samples were collected from each flask on days 2 (10 ml), 4 (10 ml), and 6 (5 ml) using a sterile pipette and filtered onto A/E filters for chlorophyll *a* analysis. Chlorophyll *a* was determined fluorometrically after 24 hr cold extraction in 10 ml of 95% ethanol [51]. Population growth rates (*r*) were calculated by fitting linear regressions to the natural log of chlorophyll *a* (*C_t_*) versus time and calculating the regression slope: ln*C_t_* = ln*C_0 _*+* rt*. Although cell quotas of chlorophyll *a* can vary over time in batch culture, changes usually manifest as cultures approach stationary phase [52]. Given that populations were growing exponentially throughout the experiment, variation in chlorophyll *a* should not markedly influence our estimates of growth rate. ANOVA was used to determine if mean growth rates varied between the two methodological techniques (flasks vs. chambered slides) across the five *M. aeruginosa* genotypes.

Relationships between growth rate and initial colony size were examined via linear regression using initial equivalent spherical diameter (ESD = (6*V*/π)^1/3^) as the independent variable. The ESD is a commonly-used metric of size for variable-shaped phytoplankton [53], [54], [55] and served to linearize the relationships. Previous studies and work in our labs have shown near-linear relationships between colony ESD and cell number [56], [57], [58] for *M. aeruginosa*. Initial colony size was used for statistical analyses, rather than colony size averaged over the incubation interval, because we wanted to avoid contaminating the independent variable (colony size) with parameters that could be influenced by the dependent variable (growth rate). Time-averaged colony size is influenced by final colony size, which can be influenced by growth rate (all else being equal, fast-growing colonies will be larger at the end of the incubation than slow-growing colonies). A homogeneity of slopes test was used to determine if the relationship between colony size and growth rate differed across genotypes of *M. aeruginosa* (i. e., genotype x colony size interaction). If the interaction was not significant, we then used an ANCOVA to test for differences in growth among genotypes that were independent of size. We also examined relationships using initial colony volume (rather than ESD) as the independent variable, in order to compare our results to the broader mass-based allometric literature, since volume scales linearly with mass.

## Results

**Figure 2:**
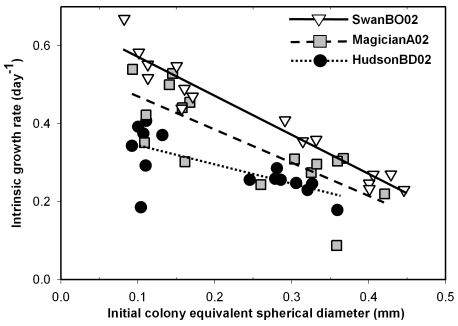
Patterns between *Microcystis aeruginosa* colony growth rate and size. Relationship between growth rate (day^−1^) and initial colony equivalent spherical diameter (ESD, mm) measured between days 1 and 8 for individuals of three *M. aeruginosa* genotypes (HudsonBD02, MagicianA02, and SwanBO02) grown in chambered microscope slides.

As expected, we found strong negative relationships between growth rate and initial colony size (ESD) for all three *M. aeruginosa* genotypes in the first experiment (*P*≤0.005, Fig. 2, Table 1). Interestingly, the slopes of the relationships varied significantly across genotypes (genotype x colony size interaction *P*<0.05), with the Hudson genotype having a slope that was roughly half of that for the other two genotypes (Table 1).

**Table 1:** Summary statistics for relationships between *Microcystis aeruginosa* colony growth rate and initial equivalent spherical diameter.

Exp	Genotype	Min ESD (mm)	Max ESD (mm)	Slope	Slope SE	Constant	Constant SE	*P*-value	Predicted *r* at 0.2 mm ESD	Predicted *r* at 0.2 mm ESD SE
1	HudsonBD02	0.092	0.360	−0.487	0.144	0.391	0.033	0.005	0.293	0.013
	MagicianA02	0.093	0.421	−0.851	0.185	0.552	0.048	<0.001	0.382	0.020
	SwanBO02	0.082	0.446	−1.002	0.072	0.670	0.021	<0.001	0.469	0.010
2	ALB3R708	0.059	0.174	−1.622	0.488	0.615	0.053	0.003	0.291	0.043
	Erie31F1206	0.087	0.375	−0.395	0.194	0.442	0.044	0.053	0.363	0.019
	HudsonBD02	0.058	0.325	−0.622	0.195	0.382	0.037	0.004	0.258	0.017
	SwanBS02	0.050	0.668	−0.587	0.239	0.533	0.068	0.022	0.416	0.032

Statistics for the relationship between growth rate (*r*, day^−1^) and initial colony equivalent spherical diameter (ESD, mm) for *M. aeruginosa* genotypes used in two experiments. Predicted growth rates (*r*, day^−1^) and standard errors (SE) for hypothetical colonies measuring 0.2 mm (ESD) for six *M. aeruginosa* genotypes (note that HudsonBD02 was used in both experiments) used in two experiments. Note that 0.2 mm ESD is outside the range of available data for ALB3R708. Exp = Experiment, Min ESD = minimum initial colony ESD, Max ESD = maximum initial colony ESD.

**Figure 3:**
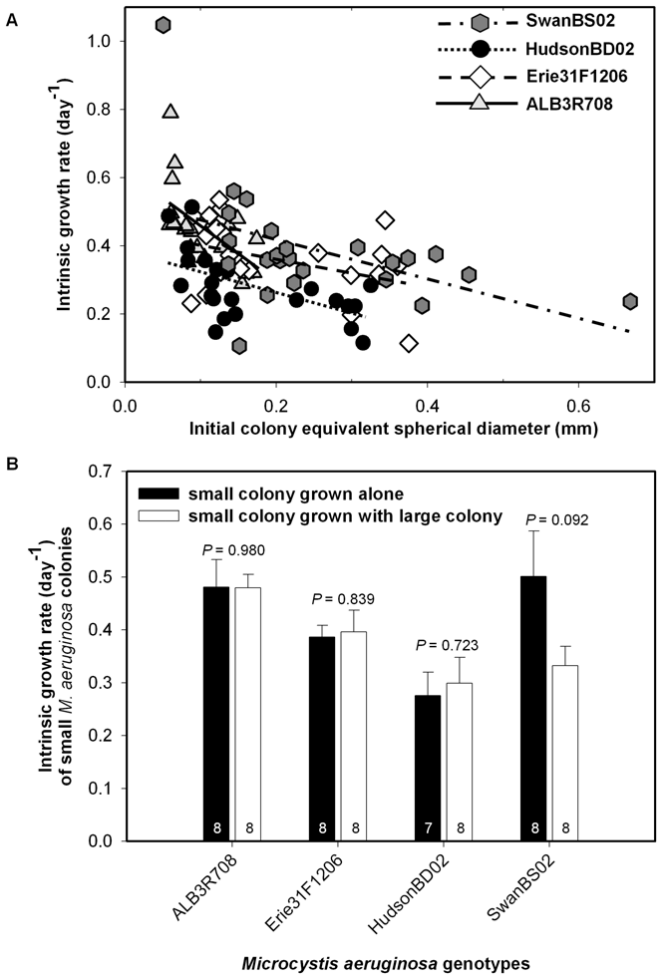
Test of colony resource exhaustion. (A) Relationship between growth rate (day^−1^) and initial colony equivalent spherical diameter (ESD, mm) between days 1 and 7 for individual colonies of four *M. aeruginosa* genotypes (ALB3R708, Erie31F1206, HudsonBD02, and SwanBS02). (B) Comparison of colony growth rates for small colonies of four *M. aeruginosa* genotypes grown in chambered slides in isolation (dark bars) or in association with a large colony of the same genotype (white bars). Error bars = 1 standard error. Inset numbers are sample sizes.

As in the first experiment, three of the four *M. aeruginosa* genotypes in experiment 2 showed significant declines in growth rate as initial colony size increased (*P*≤0.025, Table 1, Fig. 3A), with one genotype (Erie31F1206), showing a marginally significant relationship (*P* = 0.053; Table 1). Although there was a greater range in genotype-specific slopes describing the relationship between colony growth rate and initial size in the second experiment (slope range = −1.622 to −0.395, Table 1) than the first experiment (slope range = −1.002 to −0.487, Table 1), there was also twice the error around the slope for the second experiment (Table 1). Consequently, the slopes were not statistically different in the second experiment (genotype x colony size interaction term, *P* = 0.301). However, we did find overall significant effects of genotype (*P* = 0.024) and initial size (*P*<0.001) across the four *M. aeruginosa* genotypes used in the second experiment.

In the second experiment, we found no evidence of bias stemming from size-related resource exhaustion in the growth chambers. Small colonies grew similarly when grown alone or together with a large colony for all four genotypes (*P*≥0.092, Fig. 3B), indicating that large colonies did not measurably exhaust resources during the incubations. In one case (SwanBS02, which had the largest colonies, Table 1), there was a marginally significant difference in growth rate (*P* = 0.092, Fig. 3B), which may signal that we approached the limitation of the method with respect to maximum colony size (∼0.7 mm, ESD) and/or study duration (6 days).

**Figure 4:**
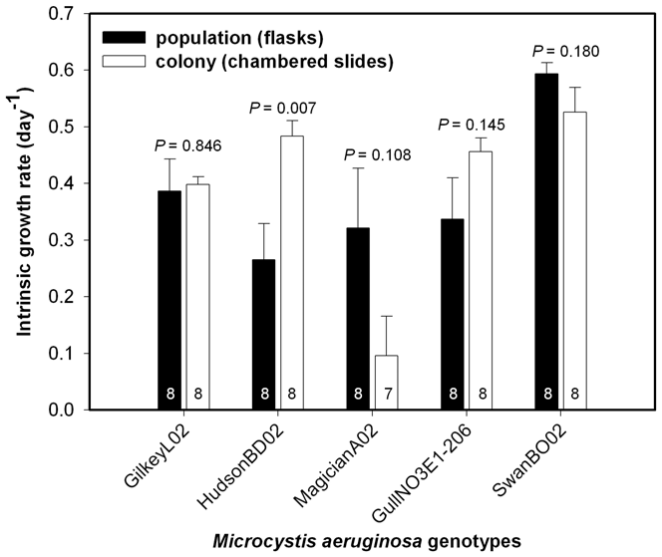
Population and colony growth rate comparison. Growth rates for five *Microcystis aeruginosa* genotypes (GilkeyL02, HudsonBD02, MagicianA02, GullNO3E1-206, SwanBO02) grown in batch culture (flasks, dark bars) versus individual colonies grown in chambered slides (white bars). Error bars = 1 standard error. Inset numbers are sample sizes.

In the third experiment, growth rates measured for individual colonies were comparable to population growth rates measured in batch cultures across five *M. aeruginosa* genotypes (growth rate mean±SE, flask = 0.381±0.057, chambered slide = 0.392±0.077, Fig. 4). There was no overall effect of method on growth rate, but there were significant differences in growth rate among the five genotypes (ANOVA, effect of method, *P* = 0.757; effect of genotype, *P*<0.001, Fig. 4) as well as a significant genotype x method interaction (*P* = 0.003). Given the latter result, we tested each genotype for differences in measured growth rates between methods. When analyzed separately, population and colony growth rates were statistically different for only one of the five genotypes tested, HudsonBD02, which grew significantly faster as individual colonies than in batch culture (ANOVA *P* = 0.007, Fig. 4).

## Discussion

We found a consistent negative influence of initial colony size on growth rate for five of six *M. aeruginosa* genotypes isolated from Michigan and Alabama lakes representing a wide productivity gradient, with only one genotype showing a marginally significant relationship (Figs. 2, 3A, Table 1). Interestingly, the relationships between colony size and growth rate exhibited a large range in slope (−1.622 to −0.395, Table 1), and in one experiment varied significantly across genotypes, within a single species. We also found that growth rate varied independently of colony size across genotype, indicating that something other than colony size affects growth rate within this single species.

With respect to methodological validation, we found no evidence of bias stemming from differential resource exhaustion by large colonies (Fig. 3B) and that the growth rates of individual colonies measured by our new method were generally comparable to population growth rates in standard batch culture (Fig. 4). In addition, for the one *M. aeruginosa* genotype (HudsonBD02) that was used in two experiments (Figs. 2 and 3A, Table 1), the slope of the growth versus size relationship did not vary significantly between experiments (experiment x colony size interaction *P* = 0.606, Table 1) despite relatively low standard errors (and so relatively high power to detect differences) for these slope estimates (Table 1). These results show that we could obtain similar responses across experiments for at least one of the tested genotypes and that the method should be valuable for other studies measuring the growth of individual microbes. However, future studies using large colonial species should consider the ratio of colony volume to medium volume in the chamber to avoid possible artifacts associated with resource exhaustion by the largest colonies. For example, the largest colony (SwanBS02) used in the first two growth experiments had an initial volume of 0.156 mm^3^ (0.000156 µl) and a final volume of 0.647 mm^3^ (0.000647 µl). The volume of the experimental medium in the chambers was 500 µl. Thus, the largest colony to medium volume ratio in our experiments was between 3.12×10^−7^ and 1.29×10^−6^ at the end of the experiment.

Also with respect to methodology, choice of experimental duration should be guided by the tension between the ability to detect changes in colony volume and the fact that growth rate variation driven by initial size will decrease over time as small colonies “catch up” in size to large colonies by virtue of their higher growth rates. For example, we found that growth rates for small colonies measured over days 1–8 were underestimated by 22% relative to growth rates calculated using data collected on only days 1 and 4 (experiment 1). Although growth-rate estimates from days 1 to 4 would provide a somewhat more accurate depiction of the relationship between growth rate and initial colony size (for example, our slopes may be somewhat underestimated), these estimates were considerably less precise than those we report over the entire interval used in the first experiment (mean squared error: days 1 to 4 = 0.0149, days 1 to 8 = 0.0036). Lower precision was likely a consequence of estimates being based on smaller changes in colony volume and only two measurements of volume. Future studies might profitably examine how experimental duration affects both the accuracy (in terms of capturing the relationship between growth rate and size) and precision of growth rates before applying the method to new questions.

We found no significant differences in growth rates for individual colonies versus populations in batch culture in four out of five cases (Fig. 4). Thus, in general, our method of measuring the growth of individual colonies compares favorably to conventional batch culture while also providing more ecological information (i.e., growth rate versus colony size). The one case where we found a difference between methods was for the Hudson Lake genotype, which grew faster as isolated colonies than in batch culture (Fig. 4). HudsonBD02 colonies grew relatively slowly in the first two experiments (Figs. 2, 3A) yet relatively fast in the third (Fig. 4), suggesting high growth variability for this genotype, in general. In any case, our results generally indicate that the method has value, and so is potentially suitable for wider applications.

We found large variation in the growth rates of individual *M. aeruginosa* colonies across six genotypes (*r* range = 0.09 to 1.05, Figs. 2, 3A, Table 1) for a range of colony sizes (ESD range = 0.06 to 0.67 mm, Figs. 2, 3A) that was similar to what we have observed in a low-nutrient lake in Michigan (Gull Lake, 2007 and 2008, ESD range = 0.03 to 0.55 mm, N = 2,775, O. Sarnelle, unpublished data). Much of the variation we observed in colony growth was likely driven by initial colony size. Such within-population variation (see also [59]) paints a very different picture of a phytoplankton population than that obtained by measuring a single, aggregate population growth rate [60].

To quantify growth rate variation that was independent of colony size, we calculated growth rates for hypothetical colonies with an ESD of 0.2 mm for each genotype from our regression analyses. For the six genotypes used in the first two experiments, these standardized growth rates varied from 0.26 to 0.47 day^−1^ (Table 1). Since this is the first study to measure the growth rates of individual colonial phytoplankters, no similar size-independent data exist for comparison purposes. Given that the size-structure of algal populations will influence interactions with competitors and herbivores, we encourage future studies incorporating the colony growth measurement technique described in this study to quantify how growth rate varies both within and across species under a variety of environmental conditions.

Given that the negative relationship between colony size and growth rate varied in slope across genotypes (Table 1), colony size clearly has distinct genotype-specific effects on colony growth rate. These findings are intriguing and could be related to intraspecific differences in colony morphology that variably conserve surface-to-volume ratios [15], [61]. For example, although the genotypes were relatively similar in shape, colonies of the Hudson genotype tended to contain more voids (i.e., regions lacking cells) than the other two genotypes used (Fig. 1), which could enhance the diffusion of solutes into and out of the colony and so mitigate the effect of increasing size [8], [61]. Data from the first experiment support this idea (i.e., HudsonBD02 exhibited the flattest slope of the three *M. aeruginosa* genotypes, Fig. 2), while data from the second experiment are less clear (i.e., HudsonBD02 had the second flattest slope of the four genotypes, Fig. 3A, Table 1). Moreover, Hudson colonies tended to have a thicker mucilaginous sheath than the other two genotypes (A. Wilson, unpublished data) and it has been hypothesized that sheaths may enhance nutrient uptake rates [62], which could again mitigate the negative effect of colony size on growth rate. However, data supporting these mechanisms are lacking and future experiments are needed to explain intraspecific variation in growth that is not attributable to size differences.

Our consistent finding that larger colonies grow slower (Figs. 2, 3) directly contradicts a past study by one of us that reported a positive relationship between population growth rate and average colony size across 19 *M. aeruginosa* genotypes in batch culture [47]. In that study however, average colony size in batch cultures was only measured at the end of the experiment, so it may be that faster growing genotypes simply grew into larger colonies in the same amount of time. In other words, that study [47] may have actually measured the effect of growth rate on final colony size. We now have evidence that genotypes vary substantially in growth rate for reasons other than size (Table 1), so this explanation is highly plausible. The technique used in the current paper is superior for assessing how colony size affects growth rate because the size of individual colonies was established prior to determining growth rate.

Our results also contrast with a recent review that failed to find a significant relationship between maximum growth rate and average colony size across many colonial green or cyanobacterial species [23]. However, a compilation of results across diverse taxa and experimental protocols will be inherently noisier and so less likely to find statistically significant relationships than a focused study of one species. Our data show quite consistently that increased colony size of *M. aeruginosa* comes at a cost of a substantially reduced growth rate (Table 1, Figs. 2, 3A) across genotypes isolated from habitats with a variety of environmental conditions. However, additional experiments are needed to determine if the observed negative relationship between colony size and growth rate holds across a wider variety of genotypes and species of colonial phytoplankton.

Given the ecological importance of phytoplankton, numerous theoretical and empirical studies have documented the physiological and ecological importance of phytoplankton size [8], [15], [17]. As in the broader literature, these studies have tended to focus on single-celled phytoplankton and are part of an important literature documenting robust allometric relationships between growth rate and phytoplankton size. Such studies have shown growth rate to be negatively related to cell size, which is expected given the functional constraints associated with large size, such as the less efficient transfer of dissolved gases and nutrients into and throughout cells [8]. To facilitate comparison of our results with these studies (presented in the form: *R* = *aM^b^* where *b* is the scaling exponent), we calculated *b* of colony growth rate versus colony volume for each genotype for the first two experiments (Table 2). We found significant negative relationships for all (*P*≤0.014) but one of the *M. aeruginosa* genotypes (Erie31F1206, *P* = 0.067) with slopes that were somewhat flatter (*b* range; –0.206 to –0.086) than what is generally reported for the log growth versus log phytoplankton cell size (–0.33 to –0.25, [15], [36], [63]) or, more generally, log body size [3]. Lower slopes for colonies may be related in part to the fact that *Microcystis* colonies are often not spherical and can be perforated, which could help to conserve surface area/volume ratio as colony size increases. In addition, a *Microcystis* colony consists of cells imbedded in a watery colonial matrix that may be less of a diffusion barrier than cell walls and cytoplasm [8]. Also, growth versus size relationships for cells are based on comparisons across taxa, whereas our results are for single genotypes, which may have an as yet undetermined influence on the slope. Finally, the slopes of the relationships between growth rate and initial colony size we report may be underestimated because our estimates of growth rate are not completely immune from the aforementioned issue of smaller colonies “catching up” in size to large colonies over the course of the incubation. In any case, our results represent the first attempt to quantify the influence of colony size on growth rate in the absence of any influences relating to taxonomic variation.

**Table 2:** Summary statistics for relationships between *Microcystis aeruginosa* colony growth rate and initial volume.

Exp	Genotype	Min colony vol (mm^3^)	Max colony vol (mm^3^)	Slope	Slope SE	Constant	Constant SE	*P*-value
1	HudsonBD02	0.000410	0.024305	−0.103	0.032	−0.810	0.084	0.007
	MagicianA02	0.000424	0.039005	−0.206	0.062	−0.979	0.147	0.005
	SwanBO02	0.000291	0.046424	−0.190	0.015	−0.838	0.037	<0.001
2	ALB3R708	0.000105	0.002759	−0.130	0.033	−0.790	0.110	<0.001
	Erie31F1206	0.000351	0.027704	−0.086	0.045	−0.680	0.117	0.067
	HudsonBD02	0.000101	0.017983	−0.140	0.039	−0.972	0.110	0.002
	SwanBS02	0.000067	0.156419	−0.124	0.047	−0.727	0.109	0.014

Statistics for the relationship between growth rate (*r*, day^−1^) and initial colony volume (mm^3^) for *M. aeruginosa* genotypes used in two experiments. Slopes, constants, and associated standard errors were calculated after log-transforming growth rate and initial colony volume to linearize the relationship. Exp = Experiment, Min colony vol = minimum initial colony volume, Max colony vol = maximum initial colony volume.

Past studies have documented the major role of body size as a driver of ecological and physiological processes. For example, small body size can enhance fitness of aquatic prey in the presence of visual predators [64]. In addition, mass-specific metabolic rate has been shown to be negatively related to size for a wide variety of organisms [3], [65]. Within this rich literature, relatively few studies have focused on the influence of body size of colonial organisms. Such studies have shown how colony size affects metabolic rate of ascidians [66], feeding rate of bryozoans [67], cnidarians [68], or sponges [69], and growth and photosynthesis of benthic cyanobacteria [32], [33] or is correlated with macronutrient content of colony-forming, marine phytoplankton [70]. These few studies have shown that universal patterns related to body size observed for non-colonial organisms generally apply to colonial plants and animals.

We have shown using several genotypes of *M. aeruginosa* from diverse habitats that colony growth rate decreases with increasing colony diameter, which appears to be the first such documentation for microscopic organisms. This cost of becoming larger should act as a counterbalance against the previously-documented advantages of larger size, such as increased resistance to grazing [25], [26] and increased migration speed [8]. Quantifying the fitness costs and benefits of changes in colony size will permit parameterization of size-structure population models for colonial phytoplankton, and so may improve our understanding of complex interactions between harmful phytoplankton, algal competitors, and grazers [71], [72], [73].
